# Correlation of TrpGly and GlyTrp Rotamer Structure with W7 and W10 UV Resonance Raman Modes and Fluorescence Emission Shifts

**DOI:** 10.1155/2012/735076

**Published:** 2012-07-22

**Authors:** Azaria Solomon Eisenberg, Laura J. Juszczak

**Affiliations:** Department of Chemistry, Brooklyn College of the City University of New York, 2900 Bedford Avenue, Brooklyn, NY 11210, USA

## Abstract

Tryptophyl glycine (TrpGly) and glycyl tryptophan (GlyTrp) dipeptides at pH 5.5 and pH 9.3 show a pattern of fluorescence emission shifts with the TrpGly zwitterion emission solely blue shifted. This pattern is matched by shifts in the UV resonance Raman (UVRR) W10 band position and the W7 Fermi doublet band ratio. *Ab initio* calculations show that the 1340 cm^−1^ band of the W7 doublet is composed of three modes, two of which determine the W7 band ratios for the dipeptides. Molecular dynamics simulations show that the dipeptides take on two conformations: one with the peptide backbone extended; one with the backbone curled over the indole. The dihedral angle critical to these conformations is *χ* ^1^ and takes on three discrete values. Only the TrpGly zwitterion spends an appreciable amount of time in the extended backbone conformation as this is stabilized by two hydrogen bonds with the terminal amine cation. According to a Stark effect model, a positive charge near the pyrrole keeps the ^1^L_a_ transition at high energy, limiting fluorescence emission red shift, as observed for the TrpGly zwitterion. The hydrogen bond stabilized backbone provides a rationale for the C_methylene_-C_*α*_-C_carbonyl_ W10 symmetric stretch that is unique to the TrpGly zwitterion.

## 1. Introduction

Tryptophan fluorescence emission is a convenient, intrinsic probe of protein environment. Thus, it is used in a wide variety of studies ranging from protein folding [1–4], structure [5, 6], and stability [2, 7, 8] to protein dynamics [5, 9] and ligand binding [10, 11]. The tryptophan emission maximum displays wide variability, anywhere from 355 nm for solvent-exposed tryptophan to 303.5 nm, for the “buried” Trp in the M97V mutant of *Rhodospirillum rubrum* dI transhydrogenase [12]. This association of solvent exposure with fluorescence emission maximum provides for a broad, qualitative assessment of tryptophan environment. However, a detailed molecular level understanding is lacking. Earlier work focused on classifying emission shift based on tryptophan exposure to water [13, 14]. More recently, electrostatic contributions to tryptophan emission shifts were calculated for both the protein matrix and water [15–17]. In this internal Stark effect model, the widely used but simplistic association of red-shifted fluorescence emission with water exposure was not found. Moreover, the protein matrix plays a critical role in organizing water molecules, and so there is a subtle interplay between solvent and protein. The resultant single data point, the fluorescence emission maximum, does not suggest this complexity.

 We apply vibrational spectroscopy to augment our understanding of Trp fluorescence emission shifts. UVRR spectra are sensitive to environment and are rich in well-resolved vibrational peaks that, unlike fluorescence, are specific to different parts of the indole ring. For example, the tryptophan UVRR W3 mode correlates with the *χ* ^2^ dihedral angle between the indole ring and the peptide backbone [18–20].

We focus our study on TrpX and XTrp dipeptides (where *X* is any amino acid) in the zwitterionic and anionic states because TrpX zwitterions consistently display an 8–10 nm, blue-shifted emission maximum relative to the other three dipeptide species [21]. These dipeptides are good models for tryptophan photophysics in proteins because the fluorescence properties of the set of rotamers for a specific dipeptide can be varied through adjustment of solution conditions [22, 23].

 In this study—the first in a series—we begin with the simplest Trp dipeptides, TrpGly and GlyTrp in their zwitterionic and anionic forms. Observed changes in the UVRR spectra of each are coupled with *ab initio* structural calculations and nanosecond molecular dynamics (MD) simulations to provide insight into the molecular level determinants of Trp fluorescence emission. Our results show that the W7 and W10 modes are sensitive to both primary structure and charge state. The W10 mode Raman shift for each dipeptide species correlates with its fluorescence emission maximum. The W7 mode Fermi doublet peak intensity ratio, *I*(1360 cm^−1^)/*I*(1340 cm^−1^) [24, 25], is also found to correlate with the tryptophan dipeptide fluorescence emission maxima in the two charged states. Thus, our study reveals tryptophan molecular vibrations that are common to tryptophan in a red-versus-blue emission state and relates this experimental information on specific rotamers to simulated molecular configuration.

## 2. Methods

Tryptophan dipeptides were purchased from Research Plus Inc. (Barnegat, NJ) and used as provided. For UVRR spectroscopy, where the signal cross-section is low and therefore relatively high tryptophan concentrations are required, all dipeptides were dissolved to a concentration of 0.3 mM. Dipeptide solubility is not an issue at this concentration as solutions of 1 mg/mL (3.6 mM) were prepared as stock solutions without issue. We do not therefore expect any oligomerization effects at 0.3 mM peptide concentration. The buffer was 1 mM sodium phosphate buffer at pH 5.5 or 9.3 to reproduce the solution conditions (and thus the fluorescence emission results) employed by Chen et al. [21].

### 2.1. Fluorescence Emission Spectroscopy

Fluorescence spectra were acquired on a PTI QM-4/206 SE spectrofluorometer (PTI, Birmingham, NJ) with an excitation of 279 nm and 1 second acquisition time for each nm step with right-angle detection of fluorescence. For emission, all dipeptide solutions were diluted to a concentration of 0.01 mM to avoid emission attenuation via inner filter effects. Within the 0.01–0.3 mM concentration range used in the experiments, we do not expect any significant oligomerization effects, and so the results for all measurements are consistent.

### 2.2. UVRR Spectroscopy

The laser source for 228.9 nm excitation was a Cambridge Laser 85-SHG continuous wave, intracavity frequency-doubled (via a beta barium borate crystal) instrument (Cambridge Laser, Fremont, CA). UV light was dispersed via a UV fused silica Pellin-Broca prism (Thorlabs, Newton, NJ) in order to spatially separate out plasma emission lines [26], which were subsequently blocked by a pinhole. The laser beam was directed and minimally focused onto the sample in a backscattering configuration. Scattered Raman signal was collected by an antireflection-(AR-) coated triplet collection lens (CVI Laser, LLC, Albuquerque, NM) and focused with a 2′′ diameter focal length matched lens on the entrance slit of the monochromator. Additionally, the Raman signal is scrambled immediately before entrance into the monochromator via an AR-coated depolarizer (Optics for Research, West Caldwell, NJ).

The Raman signal was dispersed by a Horiba/Jobin Yvon model 1000 M single-grating (3600 grooves/mm, resolution 2 cm^−1^) monochromator (Edison, NJ) and collected by a liquid nitrogen-cooled Symphony charge-coupled device camera (Horiba/Jobin Yvon, Edison, NJ). The monochromator-CCD camera resolution is 0.1 Å (2 cm^−1^) as determined with a mercury-argon calibration lamp (Oriel Instruments, Stratford, CT). The UV power at the sample is 1 mW. The monochromator slit width is set to 150 microns for all measurements.

Typically, 0.5 mL of a solution sample in a 1 cm diameter quartz NMR tube is spun using a precision Raman spinner (Princeton Photonics Inc., Princeton, NJ) and rastered using a small DC motor mounted on a lab-assembled stage to prevent UV-induced sample damage.

The digitalized signal is saved on a desktop computer using the Synergy software program, an Origin 7.5-based program (OriginLab Corp., Northampton, MA) provided by Horiba/Jobin Yvon. For the serial acquisitions, the first is compared to those taken at the end. If spectral changes are found, end acquisitions are rejected. Raman shifts are calibrated with peaks from toluene and ethanol. The Origin 7.5 application was used for all spectral processing and manipulation.

## 3. Computational Methods

### 3.1. DFT PCM Modeling

 Calculations on all species were executed on a single-processor Windows desktop computer using Gaussian 09 software [27] with the B3LYP method and the 6–311 (d,p) basis set [28]. Most of the relevant calculations used the polarizability continuum model (PCM) in order to include the effects of a water solvent. Gas-phase calculations of the zwitterionic forms are not possible as energy minimization attempts on the zwitterion result in a charge transfer and dissociation of the molecule; neutral species are used instead. Vibrational frequencies were scaled by a factor of 0.9614 [28]. Discrepancies between calculated and observed frequencies were accepted up to ±34 cm^−1^ as seen in the literature [28]. Each frequency was given a 20 cm^−1^ broadening to more accurately reflect the true spectra.

### 3.2. Molecular Dynamics

Gromacs simulations [29] were done on TrpGly and GlyTrp dipeptides, each in the zwitterionic and anionic state in a water solvent cubic box with a minimum of 1 nm distance between the box wall and any atom of the peptide. The suggested TIP-4p water model was used in the simulations, with ~1000 solvent molecules in each simulation. The optimized potentials for liquid simulations all atom (OPLSA) force field was used in all cases. A steepest-descent algorithm was used to minimize the energy of the structure before any simulations were performed. The simulations all took place at 298 K and used periodic boundary conditions with a modified Berendsen thermostat. For smoothness of viewing, the time step was chosen as 0.5 femtoseconds. Due to the subnanosecond relaxation time for a system of this size, we have found 2 ns to be a sufficient time for the length of most of our simulations [30–32], though to ensure clarity of some results, we have extended the simulations up to 10 ns. A complete list of all parameters is available upon request. Analysis of the molecular dynamics trajectories was done using freeware, gOpenMol [33] (CSC-IT, Center for Science, Ltd., Finland).

### 3.3. Molecular Imaging

The molecular images in Figure 2 were modeled with the freeware programs, ChemSketch and 3D Viewer (ACD/Labs, Advanced Chemistry Development, Inc., Toronto, Canada). The molecular images in Figure 9 were created with the Chimera software, beta version 1 built 1951 2004/05/11 (University of California, San Francisco, CA).

## 4. Results

**Figure 1:**
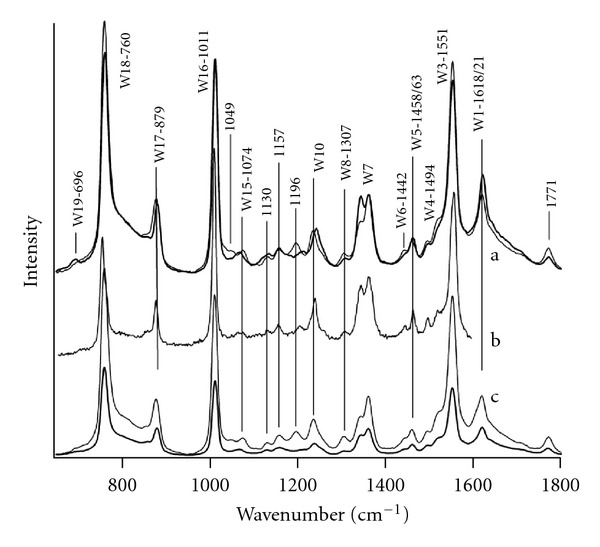
UVRR (229 nm excitation) results for tryptophan and glycine dipeptides in the anionic and zwitterionic state from 600–1800 cm^−1^: (a) TrpGly (bold line) and GlyTrp (solid line) at pH 5.5, (b) L-tryptophan in deionized water, and (c) TrpGly (bold line) and GlyTrp (solid line) at pH 9.3.

**Figure 2:**
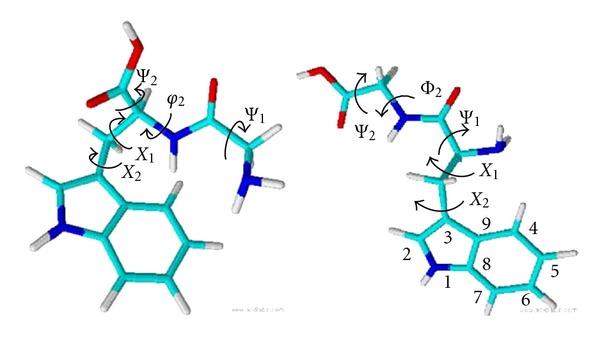
Molecular structures with dihedral angles, *χ* ^1^, *χ* ^2^,   *ψ* ^1^, *φ* ^2^, and *ψ* ^2^, defined for GlyTrp, left, and TrpGly, right. The indole ring atoms are enumerated on the TrpGly structure for reference.

UVRR spectra for the four Trp dipeptides are given in Figure 1(a) and (c). The UVRR spectrum for aqueous L-tryptophan is given in Figure 1(b) for comparison. These results reveal that intense peaks, such as W16 and W18, show little or no change in Raman shift with pH or amino acid order. In particular, the W3 band position, shown to track with the tryptophan dihedral angle, *χ* ^2^ [18–20], is at 1551 cm^−1^ for all dipeptide species. Dihedral angles are defined in Figure 2. The data in Figure 1 show that the spectral features of two other modes, W10 (1335–42 cm^−1^) and the W7 Fermi doublet (ca. 1340 cm^−1^ and 1360 cm^−1^), change with both pH and tryptophan position. Gaining a molecular level understanding of these two modes is the focus of the remainder of the paper.

### 4.1. Wavenumber Position of W10 Peaks Track with Emission Maxima for Tryptophan Dipeptides

**Figure 3:**
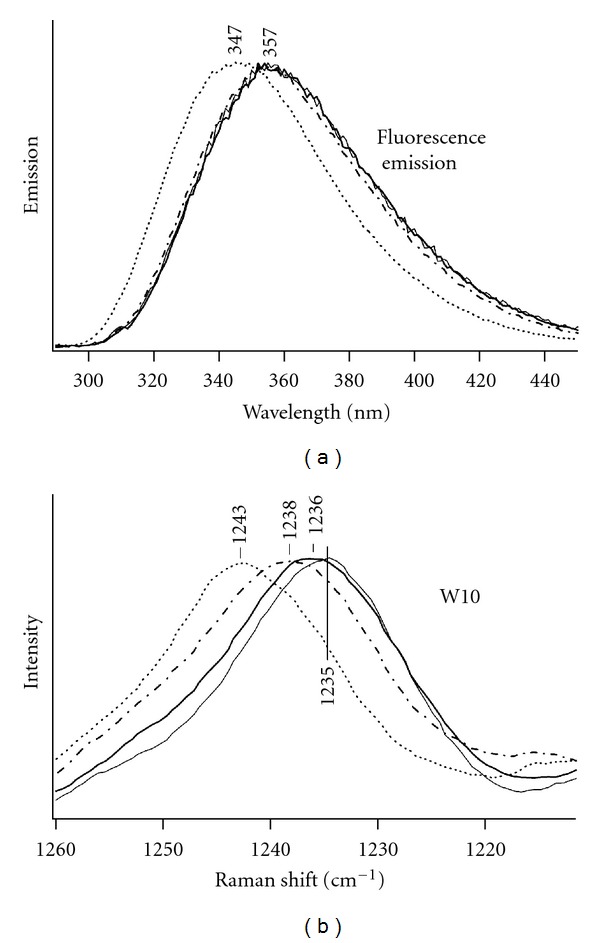
Spectra for TrpGly at pH 5.5 (dotted line) and pH 9.3 (dot-dash line), and GlyTrp at pH 5.5 (solid line) and pH 9.3 (bold line). (a) Fluorescence emission spectra, 279 nm excitation, 2 nm slits, 1 sec acquisition time, and 1 nm step size. The GlyTrp emission spectra are normalized to those for TrpGly. (b) UVRR W10 band.

**Figure 4:**
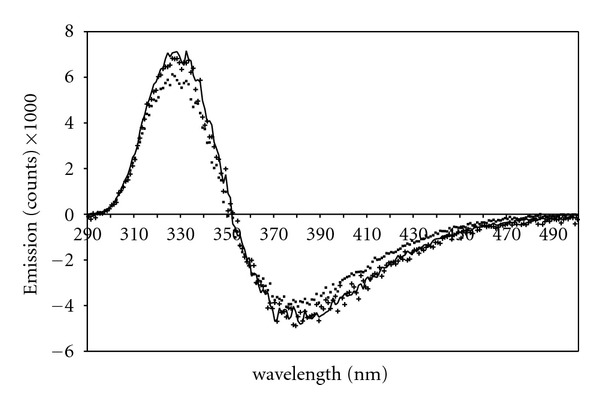
Fluorescence difference emission spectra (all spectra normalized at their emission maxima) between dipeptides: WG pH 5.5 minus: WG pH 9.3 (solid line), GW pH 5.5 (cross points), GW pH9.3 (square points). Peaks are located at 329 and 380 nm.

The normalized fluorescence emission spectra for all four tryptophan dipeptide species are given in Figure 3(a). The TrpGly zwitterion has the highest energy fluorescence emission maximum at 347 nm, (Figure 3(a)). The emission maxima of the remaining three dipeptide species are shifted to lower energy, 357 nm. Emission difference spectra between the spectrum for the TrpGly zwitterion and the spectra for all other dipeptide species are given in Figure 4. These difference spectra are similar; each is characterized by a positive band centered at ca. 328 nm and a negative peak at 380 nm. The results suggest a distinct set of conformers for the TrpGly zwitterion that is responsible for the blue-shifted fluorescence emission.

The W10 peaks for the four tryptophan dipeptide species (Figure 3(b)) parallel the trend observed in the fluorescence emission spectra (Figure 3(a)). The magnitudes of the shifts are also in parallel (Table 1). As for the set of fluorescence emission spectra, the TrpGly zwitterion is the species with highest energy W10 peak (1243 cm^−1^). A similar pattern of W10 band shifts has been found for dipeptides of tryptophan with leucine or glutamine, as summarized in Table 2. The fluorescence emission maxima for the leucine and glutamine dipeptide species are analogous to those for the tryptophan/glycine dipeptides given here (data not shown) and to those given in Chen et al. [21]. The observation of these spectroscopic trends for additional dipeptides eliminates the possibility that the W10 pattern of peaks is unique to dipeptides with glycine. 

### 4.2. The W7 Fermi Doublet Intensity Ratio, * I *(1360 cm^−1^)/ * I * (1340 cm^−1^), Tracks with Fluorescence Emission Maxima for Tryptophan Dipeptides

 The W7 Fermi doublet band intensity ratio has previously been identified as a marker for indole ring hydrophobicity, with an *I*(~1360 cm^−1^)/*I*(~1340 cm^−1^) ratio >1 for indoles in solution with aromatic and saturated hydrocarbon solvents under nonresonant Raman conditions [24]. This relationship has been applied under resonance Raman conditions as well [25, 34–39]. Additionally, large changes in the Fermi doublet band intensity ratio have been observed at tryptophan derivative pK_a_s; a ratio value ~1 is found for tryptophan at pH 6.15, and >1 at pH 13 under nonresonant Raman conditions [24]. We obtain values greater than unity for the Fermi doublet intensity ratios for three out of four tryptophan dipeptide species, as given in Table 1. The W7 ratio for the TrpGly zwitterion is again the exception. This trend is observed for the W7 intensity ratios of tryptophan dipeptides of leucine and glutamine as well (data not shown).

### 4.3. Theoretical Calculations

**Figure 5:**
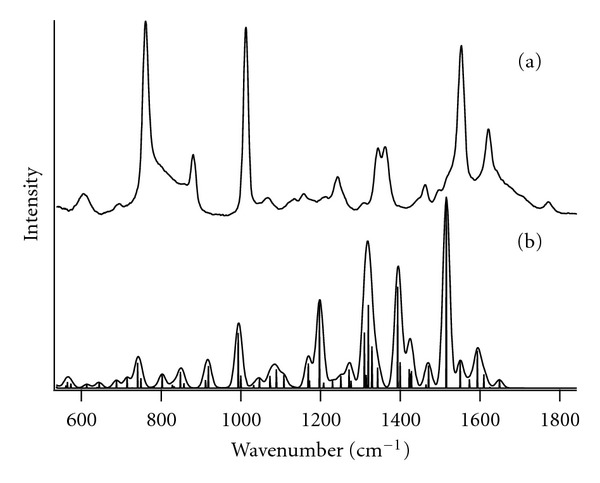
(a) Experimental UVRR spectrum for TrpGly zwitterion. (b) Calculated UVRR spectrum for TrpGly zwitterion, B3LYP DFT functional with 6–31++G** basis set, 0.9635 scaling factor (vertical lines), and with 20 cm^−1^ peak broadening (solid, continuous line).

Theoretically derived UVRR spectra provide the molecular modes associated with individual vibrations and thus aid in understanding of band changes for different dipeptide species. Calculated UVRR results for the TrpGly zwitterion along with a 20 cm^−1^ broadened representation are shown in Figure 5(b). Experimental UVRR results for the TrpGly zwitterion are given again in Figure 5(a) to illustrate relative band positions. We note that calculated bands at 1320 cm^−1^ and 1395 cm^−1^ (Figure 5(b)) are within the accepted ±34 cm^−1^ error range for band assignment to experimental peaks at 1344 cm^−1^ and 1362 cm^−1^ (Figure 5(a)), as given in the literature [28]. Comparison of calculated and experimental W7 and W10 modes is detailed below. We note that other groups have found more than one W7 fundamental in their calculations [20, 40–43]. A multiplet of bands—three to four—in the W7 region for tryptophan cation, zwitterion, and anion under nonresonance Raman conditions and in the presence of water and heavy water has also been observed experimentally [24].

### 4.4. Calculated W10 Mode

**Figure 6:**
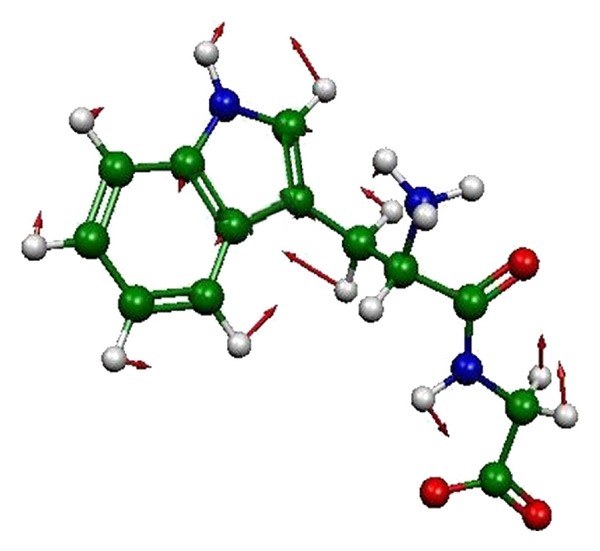
TrpGly zwitterion calculated vibrations at 1271 cm^−1^. Prominent motions for all dipeptide species include a C8-N1-C2 symmetric stretch synchronous with a C3-C9 stretch. A C_methylene_-C_*α*_-C_carbonyl_ symmetric stretch is found only for the TrpGly zwitterion. Hydrogen bending motions are found on C2, C4–7, and C_methylene_.

 DFT PCM modeling yields bands at 1262–1271 cm^−1^ that can be assigned to the W10 mode for the Trp dipeptide species (Table 1). The calculated W10 band shifts track with the experimental W10 shifts for all dipeptide species with the exception of the GlyTrp anion. Calculated vibrational movements for W10 are shown in Figure 6, and details of the atomic movements are included. The atomic displacements for the indole residue agree with those published elsewhere for 3-methylindole [44]. The W10 vibrational modes for all dipeptide species are primarily ring breathing modes centered on the pyrrole ring. One of the noteworthy vibrational differences between the W10 modes for the different dipeptide species is the tryptophan C_methylene_-C_*α*_-C_carbonyl_ symmetric stretch that is unique to the TrpGly zwitterion.

### 4.5. Calculated W7 Modes

The calculated spectra of the TrpGly and GlyTrp dipeptide species reflect the intensity changes that occur in the W7 doublet. It is imperative to understand that we are not matching calculated nonresonance Raman band intensities to resonance Raman experimental peaks. Instead, we are assigning individual calculated modes to experimental peaks. In some cases, more than one calculated mode or frequency is assigned to the same peak. The calculated bands that relate to the experimental W7 1362 cm^−1^ and 1344 cm^−1^ peaks are found at 1395 cm^−1^ and 1320 cm^−1^, respectively (Figure 5(b)). For clarity in discussion, we shall hereafter refer to the experimental W7 bands as W7a (1344 cm^−1^) and W7b (1362 cm^−1^). The calculations predict that the W7a peak, which is more intense for the TrpGly zwitterion, is composed of more than one vibrational frequency, while the W7b band is mostly associated with a single vibration. The movements of the W7b vibration on the indole ring are the same for all the species and consist of asymmetric stretches spread over the indole ring and attached methyl group, as shown in Figure  S1 (See Figure  S1 in Supplementary Material available at doi:10.1155/2012/735076). The similarity of the calculated vibrations for all the dipeptide species suggests that the W7b peak is not responsible for the observed W7 intensity ratio changes.

The calculated 1320 cm^−1^ band, corresponding to W7a, is composed of three vibrational energy transitions at 1310 cm^−1^, 1320 cm^−1^, and 1329 cm^−1^. The vibrations for these three transitions in the TrpGly zwitterion are illustrated in Figures  S2–S4. Specific atom motions common to all dipeptide species are given in caption. The 1320 cm^−1^ and 1329 cm^−1^ vibrations are similar for all dipeptide species. However, the 1310 cm^−1^ vibration does not occur in the TrpGly anion or the GlyTrp zwitterion calculated spectra. The lack of this vibration causes the calculated 1320 cm^−1^ band, which is the sum of the three transitions, to have a lower intensity for the TrpGly anion or the GlyTrp zwitterion (data not shown) and thus correctly predicts the lowered intensity of their W7a band in the experimental UVRR spectra. Even the GlyTrp anion, for which the 1320 cm^−1^ calculated band has only a slightly lowered intensity, does not have all the transitions. The calculations therefore provide an explanation for the observed lower intensity of the W7a band for both anionic species and the GlyTrp zwitterion (Table 1).

### 4.6. Molecular Dynamics Simulations

We have carried out simulations of the zwitterionic and anionic TrpGly and GlyTrp dipeptide conformations in a cavity filled with ~1000 water molecules for a period ranging from 2 to 10 ns in order to ensure that all conformational angles were sampled. Measurements of the *χ* ^1^, *χ* ^2^, *ψ* ^1^, *φ* ^2^, and *ψ* ^2^ angles, illustrated in Figure 2, were taken throughout the trajectory.

**Figure 7:**
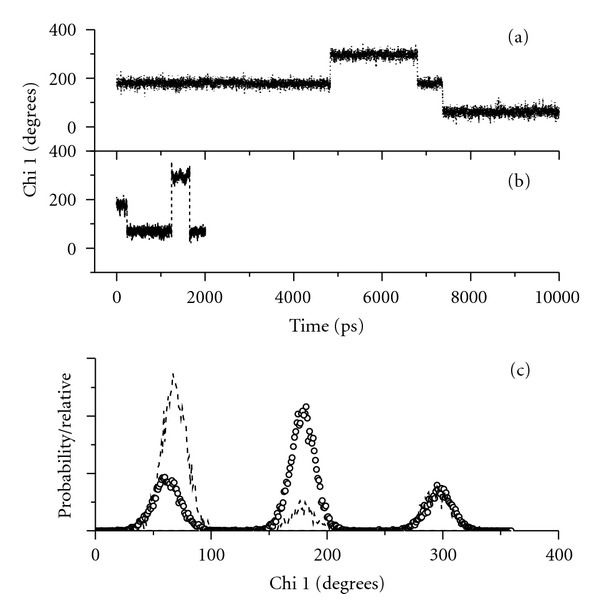
*χ* ^1^ molecular dynamic trajectories and probabilities for TrpGly species: (a) zwitterionic, 10 ns duration, (b) anionic, 2 ns duration. (c) Corresponding *χ* ^1^ probability for TrpGly zwitterion (dotted line) and anion (dashed line).

**Figure 8:**
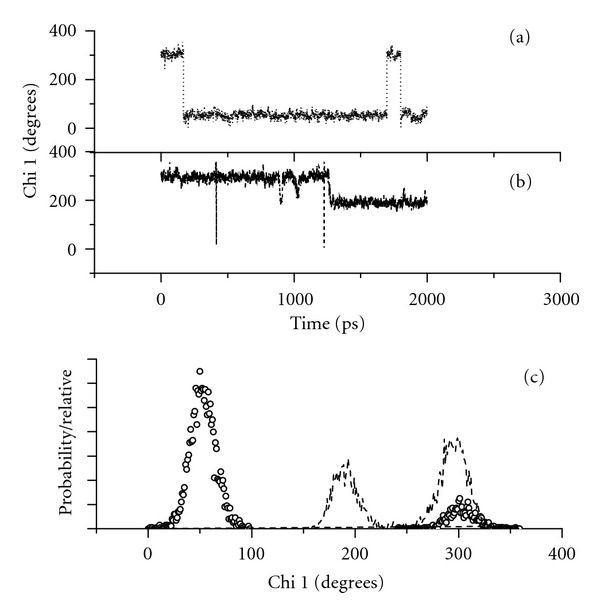
*χ* ^1^ molecular dynamic trajectories and probabilities for GlyTrp species: (a) zwitterionic, 10 ns duration, (b) anionic, 2 ns duration. (c) Corresponding *χ* ^1^ probability for GlyTrp zwitterion (dotted line) and anion (dashed line).

Two conformations predominate for all four species, and these are almost entirely dictated by the *χ* ^1^ angle. For all the species, there are only 3 ranges for the *χ* ^1^ angle: 60 ± 30°, 180 ± 25°, and 300 ± 35°, as illustrated in the *χ* ^1^ probabilities in Figures 7(c) and 8(c). MD trajectories for the TrpGly species are given in Figure 7(a) and (b) while those for GlyTrp species are given in Figure 8(a) and (b). These MD trajectories show that the conformers spend an appreciable amount of time at any one of the three *χ* ^1^ angles before switching “instantaneously” to another angle.

**Figure 9:**
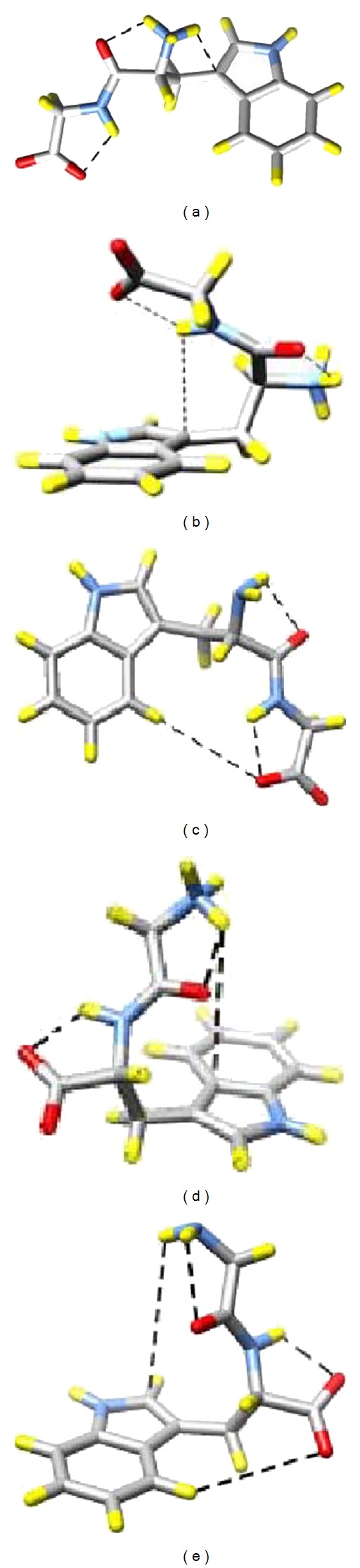
Lowest-energy molecular conformers for tryptophan dipeptides in the zwitterion and anionic forms. (a) TrpGly, pH 5.5, stretched-out backbone conformer, (b) folded backbone conformer, (c) TrpGly, pH 9.3, (d) GlyTrp, pH 5.5, and (e) GlyTrp, pH 9.3. Atoms are color coded as follows: N, blue; O, red; H, yellow; C, grey. Dashed lines indicate electrostatic interactions discussed in the text.

When the *χ* ^1^ angle is in either of the last two ranges, given above, the molecule is in its backbone “stretched-out” conformation, as illustrated in Figure 9(a). When the *χ* ^1^ angle is close to 60°, however, the molecule is in its backbone “folded” conformation, as illustrated in Figure 9(b). A set of dihedral angles (*χ* ^1^, *χ* ^2^, *ψ* ^1^, *φ* ^2^, *ψ* ^2^) for the calculated, low-energy conformers of the tryptophan dipeptide species is given in Table  S1. Note that the values of the dihedral angle, *χ* ^2^, are similar—ranging from 80° to 110°. These calculated *χ* ^2^ angles are in rough agreement with the 95° angle predicted by the W3 position at 1551 cm^−1^ [20].

## 5. Discussion

In the following paragraphs, we will describe the quantum mechanically determined low-energy tryptophan dipeptide conformers and discuss their conformational motions as determined from the molecular dynamics simulations. Afterwards, the spectral consequences of the dominant, extended conformation of the TrpGly zwitterion, pH 5.5 species, which places the terminal amine cation within hydrogen bonding distance of a pyrrole ring carbon, will be discussed.

### 5.1. Two Conformers for the TrpGly Zwitterion

 The TrpGly zwitterion spends proportionally greater time in the extended backbone conformation as shown by the *χ* ^1^ probability plot in Figure 7(c), dotted line. The extended conformation is represented by the sum of *χ* ^1^ probabilities at 180° and 300°. The simulations show that the TrpGly anion (Figure 7(c), dashed line) and the GlyTrp zwitterion (Figure 8(c), dotted line) spend some time in the stretched conformation, but neither spends as much time in the stretched conformation as the TrpGly zwitterion. The energy-minimized structures for both TrpGly zwitterion conformations are illustrated in Figures 9(a) and 9(b). In one conformer, the peptide backbone is extended (Figure 9(a)), and the N-terminal amine cation is within hydrogen bonding distance (1.96 Å) of the oxygen of the peptide bond carbonyl. Additionally, a second hydrogen on the terminal amine cation is 2.44 Å from C_3_ of the pyrrole ring. The short distance—2.44 Å—between the amine proton and the C_3_ of the indole ring does not allow for an intervening, hydrogen bonded water molecule. This close approach of an amine proton to the indole ring is unique to this conformer of the TrpGly zwitterion and to these TrpGly/GlyTrp dipeptides.

The second TrpGly zwitterion conformer, illustrated in Figure 9(b), is characterized by a backbone which folds back over the indole ring. This conformer has a slightly higher energy than the stretched conformer. There are two intrabackbone hydrogen bonds: between the terminal amine cation and the adjacent peptide carbonyl (1.95 Å) and between the C-terminal carboxyl anion and the peptide amine (2.02 Å). The peptide amine hydrogen is positioned 2.92 Å above the indole C_3_, and the carboxylate oxyanion is 4.03 Å above the indole C_8_, allowing for an intervening water molecule and hydrogen bonding. Significantly, the terminal amine cation is positioned well away from the indole ring for this conformer.

### 5.2. The TrpGly Anion Lacks Amine Interaction with the Indole Ring

The TrpGly anion spends appreciable time in a conformer where *χ* ^1^ ~ 60° (Figure 7(c), dashed line). A representative conformer, where the backbone is folded, is shown in Figure 9(c). This conformer is characterized by two intrabackbone hydrogen bonds: between the N-terminal amine hydrogen and peptide bond carbonyl oxygen (2.40 Å) and the peptide bond amine hydrogen and the C-terminal carboxylate (2.06 Å). The latter hydrogen bond provides for a folding back of the peptide backbone such that the C-terminal carboxylate partakes in an edge-on, CH–O weak interaction with the benzyl ring C_4 _(3.89 Å) [45, 46]. We note that the hydrogens of the terminal, neutral amine are turned away from the indole ring.

### 5.3. The Terminal Amine Cation of the GlyTrp Zwitterion Is Positioned above the Indole Ring

When the amino acid positions are switched, a folded-back peptide backbone provides for optimal positioning of the amine cation above the indole ring but not in close proximity. The molecular conformer for the GlyTrp zwitterion is shown in Figure 9(d). This species spends most of its simulation time in the conformation where *χ* ^1^ ~ 60° (Figure 8(c), dotted line). The distance between one hydrogen of the amine cation and C_9_ of the indole ring is 4.80 Å, well within the 6 Å cutoff for cation-*π* interactions [47]. A chain of electrostatic interactions—from amine hydrogen to water molecule to indole ring **π** electrons—is envisioned. The same hydrogen of the amine cation is also within hydrogen bonding distance (1.84 Å) of the peptide carbonyl oxygen. This hydrogen bond orients the amine proton toward the indole *π* electrons.

### 5.4. The Terminal Amine of the GlyTrp Anion Is Well above the Indole Plane

 The conformers for the GlyTrp anion are not readily described with just the *χ* ^1^ dihedral angle. This is due to the unusual variability of the *χ* ^2^ angle that the GlyTrp anion exhibits. Whereas the other three species' *χ* ^2^ angle only varies between 60° and 100°, the GlyTrp anion *χ* ^2^ angle varies between 50° and 300° and, interestingly enough, even spends a small but significant amount of time with *χ* ^2^ ~ 180°.

As for the GlyTrp zwitterion, the backbone of the GlyTrp anion, shown in Figure 9(e), folds back towards the indole ring even though the species spends no time with *χ* ^1^ ~ 60° (Figure 8(c), dashed line). The curled backbone structure (Figure 9(e)) is stabilized by two hydrogen bonds, a terminal NH to O=C peptide (2.46 Å) and a terminal C=O to HN peptide (2.10 Å) hydrogen bond. The terminal amine is also positioned above the indole plane, with an interatomic distance of 4.99 Å between one amine hydrogen and C_2_ of the indole. The terminal oxyanion is 4.03 Å from the C_4_ hydrogen.

### 5.5. Spectral Consequences of the Amine Cation-Pyrrole Hydrogen Bond in the TrpGly Zwitterion

The most striking distinction between the quantum mechanically determined low-energy tryptophan dipeptide conformers given in Figure 9 is the stretched-out conformation adapted primarily by the TrpGly zwitterion (Figure 9(a)). The resultant close approach (2.44 Å) of one terminal amine hydrogen to the pyrrole ring C_3_ can account for the relatively blue-shifted fluorescence emission maximum (347 nm) of the TrpGly zwitterion, according to an internal Stark effect model [16, 17]. The hybrid quantum mechanical-molecular dynamics studies of Callis and coworkers [16, 17] predict that a positive charge placed near to the pyrrole half of the indole or a negative charge near the benzyl ring will result in an emission blue shift. Oppositely placed charges are predicted to result in an emission red shift. A potential difference across the long axis of the indole ring determines the emission wavelength, and orientation of the charge relative to the ring is critical. Just in terms of order of magnitude, hydrogen bonding between a water molecule and the *π*-electrons of the benzyl ring of 3-methyl indole was calculated to create a red shift of 15 nm [17], which is of comparable magnitude to the 10 nm blue shift observed for TrpGly zwitterion here.

DFT-PCM calculation of the W10 mode positions for the dipeptide species is consistent with experimental W10 wavenumbers. The position of the terminal amine cation near the pyrrole ring is expected to affect the indole ring polarizability. An exoring C_methylene_-C_*α*_-C_carbonyl_ symmetric stretch is peculiar to the TrpGly zwitterion, the species with the highest W10 wavenumber. MD simulation shows two hydrogen bonds made by the terminal amine cation, one to C3 of the pyrrole ring, the other to the oxygen of the peptide bond carbonyl (Figure 9(a)). These H-bonds stabilize the orientation of the C_methylene_-C_*α*_-C_carbonyl_ segment, which is responsible for the W10 symmetric stretch that is unique to the TrpGly zwitterion.

Nonresonance Raman results for aqueous GlyTrp dipeptide show that pH titration of only the dissociable group nearest to the indole affects the solvent-sensitive Fermi doublet W7 band ratio [24]. Under our UV resonance Raman conditions, we do not observe changes in the Fermi doublet W7 band ratio for the GlyTrp dipeptide (Table 1). As for the fluorescence emission maximum and the W10 band peak position, the TrpGly zwitterion is the dipeptide species that exhibits an extreme value for the W7 band ratio (Table 1).

## 6. Conclusions

The fluorescence emission maxima shifts for TrpGly and GlyTrp zwitterionic and anionic dipeptides are matched by calculated and experimental shifts in the UVRR W10 peak and changes in the W7 Fermi doublet band intensity ratio, I(1360 cm^−1^)/I(1340 cm^−1^). This spectral correlation has not been previously realized. The W10 vibration of the TrpGly zwitterion has an additional exo-ring symmetric stretch and has the highest energy W10 vibration of all dipeptide species. *Ab initio* calculations for the 1344 cm^−1^ band of the W7 Fermi doublet reveal three modes whose intensity or position varies with dipeptide primary sequence and pH. Specifically, calculated 1310 cm^−1^ and 1320 cm^−1^ modes are diminished in intensity or absent for all but the TrpGly zwitterion, the species with the lowest W7 band intensity ratio. In nanosecond molecular dynamics simulations of these dipeptides, two conformations predominate: one with the peptide backbone extended and the other with the backbone curled back towards the indole. The significant dihedral angle in these conformations is *χ* ^1^, which takes on three angles centered at 60°, 180°, and 300°. The TrpGly zwitterion is the only species that spends appreciable time in the extended conformation. A significant molecular feature of this conformation is the position of a terminal amine hydrogen 2.44 Å from the pyrrole ring C_3_. A second terminal amine hydrogen bonds to the peptide carbonyl, securing the alignment of the C_methylene_-C_*α*_-C_carbonyl_ of the indole ring. This hydrogen bond reinforcement of the extended backbone may be responsible for the exo-ring C_methylene_-C_*α*_-C_carbonyl_ W10 symmetric stretch that is peculiar to the TrpGly zwitterion. The effect of the amine cation position on the fluorescence emission maximum for the TrpGly zwitterion is predicted by a Stark effect model [48]. This model predicts a fluorescence emission shift to higher energy when a positive charge is near the pyrrole ring. This blue shift is observed experimentally for the TrpGly zwitterion where MD simulation shows that a hydrogen from the terminal amine cation is positioned 2.44 Å from the indole ring C_3_. UVRR and fluorescence spectroscopic studies of other tryptophan dipeptides are underway. Similar fluorescence emission shifts have been found for other tryptophan dipeptides, but the specifics of backbone-indole ring interaction are unexplored [21]. With the insight offered by UVRR spectroscopy, molecular dynamics simulation, and employing the Stark effect model, we will continue to map fluorescence emission shifts to specific details of molecular environment for tryptophan.

## Supplementary Material

Supplementary material consists of a table of calculated dihedral angles for the most stable tryptophan dipeptide conformers, and molecular illustrations of calculated vibrations, 1310 cm^-1, 1320 cm^-1, 1329 cm^-1 and 1395 cm^-1, for the TrpGly zwitterion. (NB: ^ indicates superscript for the -1 designation.)

## Figures and Tables

**Table 1:** Fluorescence emission maxima, experimental and calculated W10 band peaks and W7 Fermi doublet band ratio, *I*(1360 cm^−1^)/*I*(1340 cm^−1^), for GW and WG dipeptides at pH 5.5 and 9.3. Parenthetical values are for shifts relative to values for the WG zwitterion species.

Peptide, pH	Experimental W10 Raman Shift (cm^−1^)	Calculated W10 Raman Shift (cm^−1^)	W7 *I*(1360cm^−1^)/*I*(1340cm^−1^)	Fluorescence emission (nm)
WG, 5.5	1242	1271	1.0	347
WG, 9.3	1239 (−3)	1267 (−4)	1.4	355 (+8)
GW, 5.5	1235 (−7)	1268 (−3)	1.6	357 (+10)
GW, 9.3	1236 (−6)	1262 (−9)	1.6	357 (+10)

**Table 2:** UVRR W10 peaks (1/cm) for Trp dipeptides at pH 5.5 and 9.3.

Dipeptide	W10 wavenumber (1/cm)	
	pH 5.5	pH 9.3
GluTrp	1235	1236
TrpGlu	1241	1238
LeuTrp	1237	1237
TrpLeu	1240	1237
