# Supplementary material for: Correlation of TrpGly and GlyTrp Rotamer Structure with W7 and W10 UV Resonance Raman Modes and Fluorescence Emission Shifts
Source: J Amino Acids. 2012 Jul 22;2012:735076. doi: 10.1155/2012/735076 (PMC3408653; doi:10.1155/2012/735076)
Supplement: Supplementary file 1 — Supplementary material consists of a table of calculated dihedral angles for the most stable tryptophan dipeptide conformers, and molecular illustrations of calculated vibrations, 1310 cm^-1, 1320 cm^-1, 1329 cm^-1 and 1395 cm^-1, for the TrpGly zwitterion. (NB: ^ indicates superscript for the -1 designation.) [file 735076.f1.pdf]

Supporting Information

Correlation of TrpGly and GlyTrp Rotamer Structure with W7 and W10 UV Resonance Raman Modes and Fluorescence Emission Shifts

Azaria S. Eisenberg and Laura J. Juszczak

Table S1. Calculated dihedral angles (χ^1, χ^2, ψ^1, φ^2, ψ^2) in degrees for the most stable tryptophan dipeptide conformers

**TrpGly species**	**(χ^1, χ^2, ψ^1, φ^2, ψ^2)/degrees**
**Zwitterion (stretched)**	(-171, 108, -80, -180, 0)
**Zwitterion (folded)**	(64, 80, 155, -175, -178)
**Anion**	(158, 96, -76, -126, 1)
**GlyTrp species**	
**Zwitterion**	(61, 92, 177, 140, 13)
**Anion**	(-66, 102, 160, -131, 168)

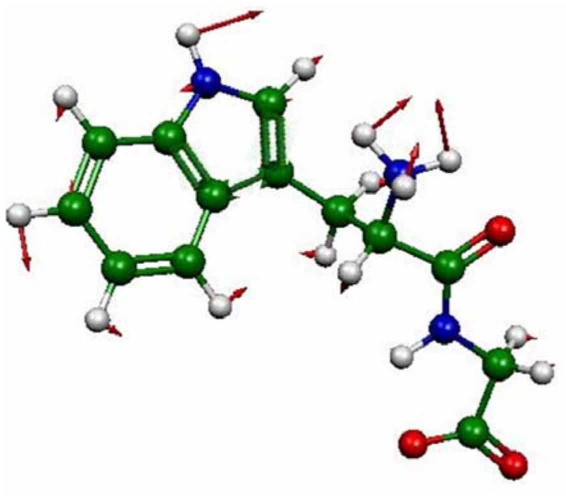

1395

Figure S1. TrpGly zwitterion, calculated vibration at 1395 cm^{-1}. These motions are common to all dipeptides: C8---N1---C2, C5---C6---C7, and C9---C1---$C_{methylene}$ asymmetric stretches and N1---H and C6---H in-plane bending.

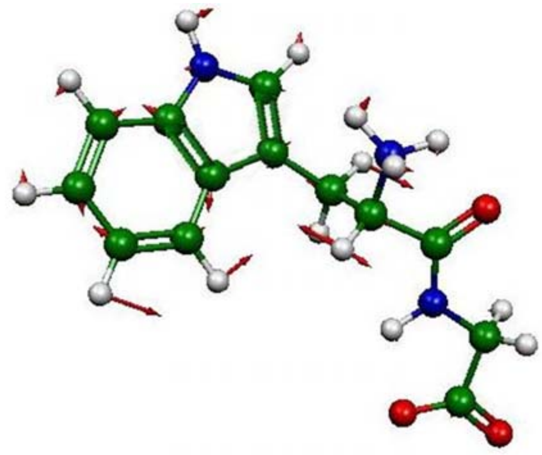

1310

Figure S2. Calculated vibration at 1310 cm^{-1} for the TrpGly zwitterion. Atom motions are similar to those for the 1320 cm^{-1} vibration.

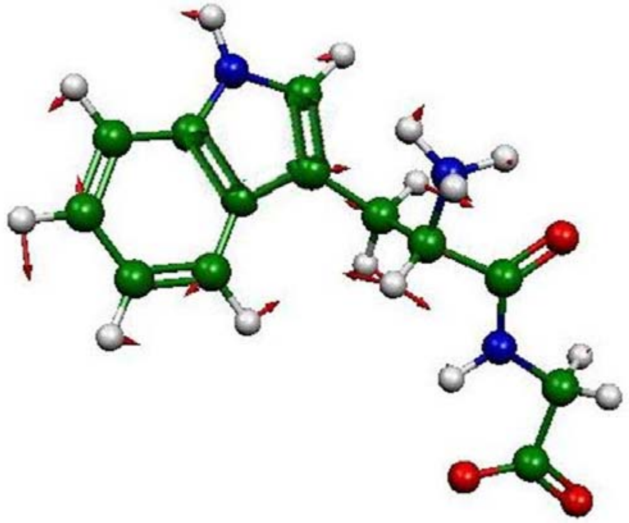

1320

Figure S3. TrpGly zwitterion, calculated vibration at 1320 cm^{-1}. This vibration contains asymmetric stretches at C8---C9---C4, C9---C3---$C_{methylene}$, and C5---C6---C7, with an N1---C2---C3 bend. Prominent off-ring vibrations are the C3---$C_{methylene}$---C_{α} asymmetric stretch, the $C_{methylene}$---H bend, and the C_{α}---H bend for all Trp dipeptides**.**

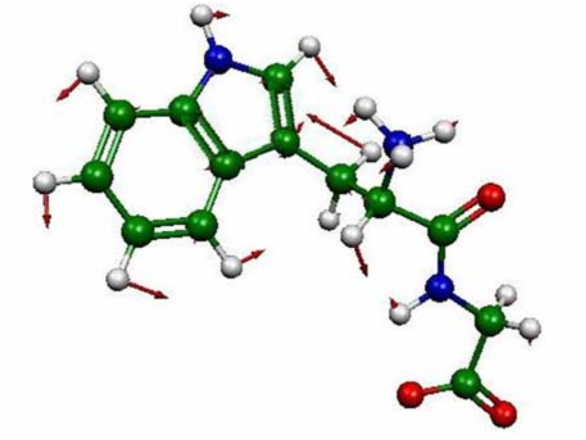

1329

Figure S4. TrpGly zwitterion, calculated vibration at 1329 cm^{-1}. Vibrations for all Trp dipeptide species consists of synchronous, C---H in-plane bending for C4 to C7, with a C2---C3---C4 asymmetric stretch as well as off-ring H-bends at $C_{methylene}$ and C_{α}. All but the TrpGly zwitterion have an additional C4---C9 stretch.
